# Self inflicted human teeth bites: a case report

**DOI:** 10.11604/pamj.2014.19.353.4561

**Published:** 2014-12-05

**Authors:** Satinder Pal Singh, Akashdeep Aggarwal, Sumeet Kaur, Dalbir Singh

**Affiliations:** 1 Department of Forensic Medicine, Post Graduate Institute of Medical Education and Research, Chandigarh, India; 2 Department of Forensic Medicine, Government Medical College, Patiala, Punjab, India; 3 Department of Biochemistry, Government Medical College and Hospital, Chandigarh, India

**Keywords:** Human bite, teeth marks, fabricated injuries

## Abstract

Human infighting has been a part of our civilization since times immemorial. These incidences may go unnoticed or may attract attention of law enforcing agencies depending upon the severity of the offence. Though weapons are generally employed to inflict injuries, rare cases have been reported in literature where human teeth have been used to serve this purpose. Human bites may be self inflicted or self suffered in connivance with others to level an allegation against an adversary. We are presenting here such a case where such injuries were produced to bring a false charge against a neighbor.

## Introduction

Infliction of injuries by means of teeth represents an aggressive human behavior that is not a commonly occurring phenomenon. The most common locations of human bites have been reported to be upper extremities followed by head & neck region [1, 2]. It has been reported in literature that human bites represent the third most common type of mammalian bites, next only to that of dogs and cats [1–3]. Other have however, reported human bites to be next only to dog bites [4]. These bites usually occur during a physical fight with an enemy. Very rarely they are produced by the complainant himself/herself or with the help of a “friendly hand”. The usual purpose is to bring a false charge against somebody or to make the genuine injuries that have been sustained during a brawl, look more serious [5]. Though both males and females can present with such injuries, males are far more frequently affected than females [2]. The most common types of this aggressive behavior are represented in the form of abrasions, contusions and sometimes lacerations [6].

## Patient and observation

**Figure 1:**
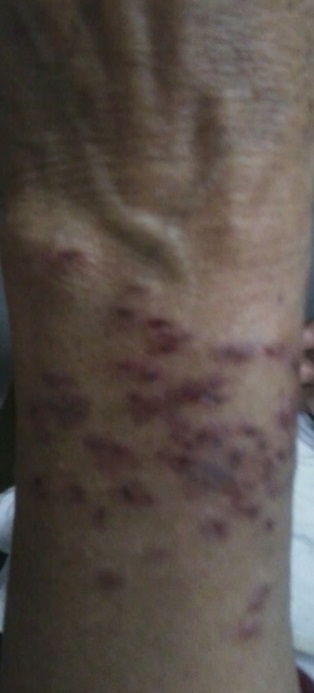
Left forearm showing human bite marks

**Figure 2:**
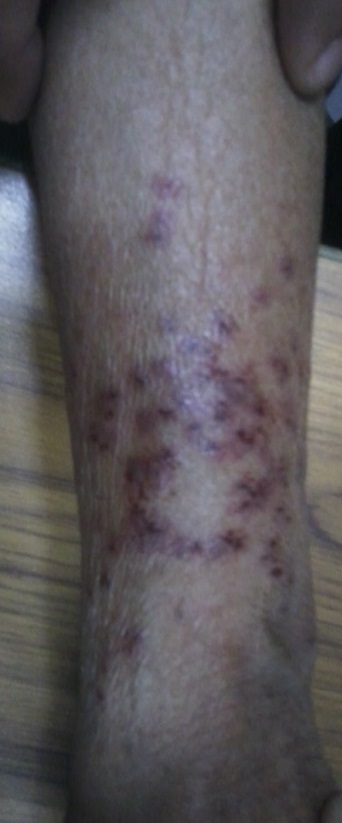
Left forearm as seen from above

A 53 year old female presented in the emergency department as a case of alleged assault. The victim was accompanied by her husband. She stated that while her husband was away for work, she had an altercation with her neighbor (a lady) regarding boundary dispute of their adjoining houses. The events took an ugly turn and they got violent. During fighting, her neighbor pulled her hair and inflicted bite marks on left forearm of the victim. A careful examination of the injuries (Figure 1) revealed the presence of numerous small “abrasions and bruises” on the dorsal aspects of left forearm in the lower two third part in an area of 12 cms x 6 cms. The size of injuries varied from 0.5 cms x 0.4 cms to 0.6 cms x 0.5 cms. Though these injuries were quite numerous and overlapping at places, an arched pattern resembling human dental curvature could be made out (Figure 2). These injuries had clearly defined margins. The surrounding skin showed reddening. No itching was present. No other injuries except complaint of pain in scalp area consistent with pulling of hair were reported. Swabs were collected from the site of injury, sealed and sent for examination for detection of saliva and possible DNA matching or detection of any irritant chemical substance. The chemical analysis report confirmed the presence of human DNA but it did not match with the sample obtained from the neighbor. A suspicion of foul play arose. Sample was then matched with the DNA of patient and a positive match was found. With the availability of chemical examination report the police thoroughly interrogated the “patient” and she admitted that the fight never took place and she and her husband had planned to get neighbors behind bars because of their unsolved boundary dispute. Though her husband participated in the planning of this conspiracy, it was only her who actually produced these injuries on herself. The couple was then booked under relevant sections of the Indian Penal code.

## Discussion

The fabricated injuries are generally in the form of superficial incised or lacerated wounds and less frequently abrasions and contusions [7]. Human bite injuries are not commonly encountered in routine medical practice. The present case is unique in the sense that these human bites were self inflicted and presented to have been received in a scuffle. The anterior teeth are involved in the production of such injuries. These injuries in the form of bruises are produced by the positive pressure that is generated when the teeth of both jaws are approximated that leads to disruption of small blood vessels of the skin that is gripped. The negative pressure as a result of suction and trusting of tongue also plays a role in this process [8].

The injuries were numerous in the above mentioned case and at some points did not even resemble with arched pattern of teeth. Therefore, it also led to a suspicion that some irritant might have been applied to produce artificial bruises. This was another point that was kept in mind while sending the samples for chemical analysis. The fact that injuries may be fabricated to simulate an offence has also been reported by other researchers [9]. In such cases, the role a careful history besides medical examination is imperative as ordinarily such “complainants” cannot satisfactorily answer all the properly framed questions (e.g. the relative position of warring persons, delay in getting medical attention and presence/absence of eyewitnesses) of the physician.

## Conclusion

The fabricated injuries generally pose a diagnostic dilemma to the inexperienced physicians [5]. This case emphasizes the need for the proper training of physicians who in general have very little exposure to medicolegal work during their graduation. In a suspected case of bites, the necessary swabs should be taken and properly preserved so that they could be matched with the alleged assailant. Failure on the part of physician to do this will not only bring a charge of negligence but also misguide the law enforcing agencies in wrong direction and miscarriage of justice. The physicians should keep in mind that even if the injuries are fabricated, they remain calm and provide adequate medical care to the patient [10]. The case also highlights the degrading moral standards of the society where a person injures himself/herself to frame a false charge against someone. Strict laws should be made for such offenders to send a strong message to society. People should also be encouraged to solve their disputes peacefully or through legal channels.
